# The Nurse and the Sick-Room

**Published:** 1887-07-02

**Authors:** 


### A Noble Profession: Nursing the Sick.

### THE NURSE AND THE SICK-ROOM.

By Miss Mollett.

The following paper was read by Miss Mollett to the members of the Hospitals Association.

Miss Mollett, who was formerly Superintendent of nursing at the National Hospital, Queen Square, Bloomsbury, and is now Matron of the Chelsea Workhouse Infirmary, had evidently bestowed very considerable thought and care upon her paper, which we are happy to reproduce *in extenso*. "The Nurse and the Sick-room" is, we understand, the first paper which Miss Mollett has ever publicly read, and we trust that the sound and practical views which it contains will not be without their influence in removing some of the errors and prejudices which still survive here and there in some of our medical charities.

Miss Mollett said: "My task to-night is to take up the thread where Miss Manson laid it down. After leading you through the past centuries in which nursing was developed, she described to you how during those centuries the profession rose and sank, and how during the last twenty years it has again gradually risen by faithful work from a condition of mere drudgery to an intelligent coadjutorship with medicine and surgery. I shall now try and describe to you the sort of woman we think a nurse should be: one who by character and training is fitted to hold one of the highest and most responsible positions a woman can hold.

"First, I should like to quote some words of Carlyle's to you, that it would be well for all nurses, as for all other workers to bear in mind, words that form the key-note of all true labour: 'All work is noble; work is alone noble, be it here said and asserted once more. Genuine work alone, what thou workest faithfully, that is eternal, as the Almighty Founder and world-builder Himself.'

"There is, I hold, no work honestly undertaken, done from honest and true motives to the very best of a man's or woman's abilities, that is not ennobling and does not raise the character of the worker, just as there is no work that is undertaken from low and selfish motives, without the true love of labour, that does not debase and degrade it. To bring the work he loves, be it ever so simple, even near the ideal he has in his mind's eye, the true worker will spare no sacrifice to make it thorough; he will carry all his personal individuality into the effort towards perfection, and that effort will be a powerful means towards cultivating the highest qualities of his character. For work to be well done, to be done only approximately to our ideal as to how it should be done, must be really loved. Nobody does his duty so thoroughly, or with such intense zest, as the man who believes that his profession or calling is the finest in the world, and the one thing for him really worth doing well, who does his work from sheer love of it, with no scamping of minor details, nor bringing to it the bare measure that is required of him, but the full force of his energies and faculties and his keenest interest.

"Now, what is true of all other professions is equally so of our own. I myself think that nursing is the finest profession in the world for women. The scope it gives to our mental and bodily powers; the manner in which it turns to account all we have ever learnt, whilst daily calling upon us to add to our knowledge, are more than sufficient to prevent the interest every intelligent woman must feel in her work from ever flagging; while the constant need we have of exercising all our innate womanly tact and refinement, our compassion and our sympathy, is quite strong enough to prevent our becoming hard, callous, or indifferent.

"I should like to state here once and for all, that for the tone that prevails in a hospital ward, both as regards the students and the patients, the nursing staff is mainly responsible. If the patients are rough, loud, disrespectful, and inclined to grumble; if there is a general tendency among the students attached to the ward not to respect the privacy, the comfort, and the feelings of the patients, it is to the sister and nurses that you must look for the explanation. Unless the head of the ward is a woman with sufficient firmness of character to carry her womanly refinement into the routine of ward work, insisting with courtesy and tac on making her influence felt in what ought to be her sphere, she is not fit to be in charge of a ward. It is not so very difficult for a clever woman to gain a sufficient smattering of medical and surgical knowledge to suffice a good nurse; but none but a really talented woman, one who not only thoroughly knows her work both practically and theoretically, but who is besides fitted both by breeding and education, to rule is fit to be a ward sister, the head to whom such a large and varied household as that which a large ward holds can look for their example and commands. Her good discipline should make smooth all angles and rough corners; her boundless patience to those in her charge, the modesty she shows with regard to her knowledge, should prevent her nurses from becoming forward; the care with which she attends to her patients' wants should disarm their grumbling; with the highest respect for the dignity and honour of her profession, her ward, its discipline, organisation, and management should occupy the foremost place in her thoughts, and it should be her effort to make it the model of a public sick-room.

"It seems almost superfluous to state again what has so often been said of the ideal nurse, of the woman whose pity and compassion must be tempered by firmness and justice, and who must above all things have learnt that most difficult lesson for a woman to learn, perfect obedience to those in authority. This point of discipline touches on a question that has often been discussed, as to whether, in thoroughly well-organised hospitals, where perfect routine is observed, the nursing has not a tendency to become hard and mechanical. Though this is undoubtedly a fault to be guarded against, it is not nearly so prevalent as some would have us believe it to be, nor are its effects nearly so baneful as is often imagined. Constant contact with anything in any occupation tends to a certain amount of familiarity with it; and a nurse who is daily and hourly engaged in tending the sick, becomes even more accustomed to their sight than the medical man who sees them only at intervals, and then for a short time. In this way a nurse certainly loses, and should lose, that exceeding susceptibility to the sight of suffering. If she is well trained she takes a more educated and scientific view of the subject. She is not only more keenly alive than the majority of women to the amount of pain actually being borne by the patient, but is mistress of the best and readiest means of relieving it, and is not hampered by a nervous dread of adding to it by ignorant handling. She is able, therefore, to perform all necessary offices without nervousness and without hesitation. A nurse who is naturally a tender-hearted woman will retain full sympathy with her patients personally; but if she is naturally hard, the routine of her work will prevent her patient from suffering in his wants from her lack of sympathy, as he might otherwise undoubtedly do. As to which will actually be the best nurse there cannot of course be two opinions; but it is not the routine of work that crushes a woman's natural sympathy. Where it really exists it only minimises the amount of harm done where it is unfortunately lacking. In those hospitals and infirmaries where nursing has not attained to the dignity of a profession, the evils attending a lack of routine and trained knowledge in nursing are very palpable.

"There the good or bad nursing depends almost entirely on the personal disposition of the nurse. Though ignorant of the routine of treatment, she may yet, with kind sympathy and perfect obedience to orders, make her patients comfortable up to a certain point; but without the knowledge of how much ought to be done for each individual, and how best to set about it, she is apt to fall into the mistake of giving more time and attention to those among her patients who appeal most directly to her personal sympathies, and scamping those who are less interesting to her, or who are more bad-tempered, disagreeable, and trying, but who have none the less an equal right to an equal share of her care and attention. Still I have seen many women, with little training, most kind, sympathetic, and conscientious nurses, and failing to carry out their duties properly only from ignorance and want of disciplined training. Where, however, an ignorant and untrained woman is wanting in sympathy and conscientiousness, the result as regards both ward and patients is very disastrous. With no machine-like habit of doing by routine what is necessary for her patients, she attends grumblingly and unwillingly to the unavoidable duties of her position, and her patients hate or fear her, if they do not despise one whose ignorance they cannot fail to remark.

"A well-trained nursing staff with a proper routine of nursing is one of the greatest boons modern progress has conferred on hospitals if they would but believe it. I lay great stress on good discipline, strict attention to rules and regulations in nursing, as being the root and marrow of the whole modern system. A large, well-managed hospital is a very complicated, beautifully jointed piece of machinery, of which every screw and nut must be in its place, or the cogs and wheels will not work properly. A nurse is not called upon to decide what her work shall be, and how she shall do it; she is called upon to obey, to obey intelligently but implicitly. Where it is necessary for a hospital nurse to use her own discretion, the discipline of an institution must be at fault, or those above her must be unfit for their posts. She should never receive orders or instructions that admit of any doubt; every eventuality should be fore seen and provided against; proper instructions should be given for every emergency. From the manner in which these instructions are carried out will the nurse's status be judged. She is responsible for the punctuality, the conscientiousness with which she obeys the matron's home regulations, the sisters' ward-rules, the doctor's instructions—she is not called upon to regulate the home or the ward, nor to treat the patients. She has voluntarily placed herself of her own free will in a subordinate position, and to fill it honestly, faithfully, and intelligently should be a point of honour with her; nor should she consider it a clever thing to evade the rules, criticise the medical staff, and, if she possibly can with impunity, use her own judgment in the treatment of cases. One of the gravest faults of the modern half-trained nurse is an overweening opinion of her own importance and capabilities. A well-trained nurse values her own position too highly, and knows her own duties too thoroughly, to make the mistake of encroaching on those that do not belong to her.

"I have often been much struck by one peculiar phase of mind in some otherwise good, clever nurses; that is, the difficulty with which they grasp the fact that a ward is a sick-room—a sick-room, it is true, on a large scale, but still to be treated on the same principles. Many nurses who, when they have a comrade off duty with, say a headache, will carefully darken her room, tread gently, shut the door carefully, and beg the other probationers to keep quiet, never seem to consider that their patients require like thought, that it is as necessary to keep a ward quiet as a sick-room, and that a fever case with a racking headache is quite as susceptible to noisy shoes, loud talking and laughing, as a nurse with neuralgia.

"I have often wondered as to how some patients ever bear the unavoidable disturbance of a scrubbing morning in those hospitals where that relic of barbarism still exists, but where there is besides a nurse who considers it rather clever to 'bustle' round, regardless of the noise she makes: the patients are indeed to be pitied. Nurses are very much inclined to fall into the error of looking at a ward too exclusively from their own point of view, and not from the patients'. A ward is a sick-room, and, to the nurse at all events, the patients are, or should be, sick men and sick women, whose nerves are as easily jarred as the nurse's own when she is ill, and who are quite as easily hurt as she would be at any unkindness, thoughtlessness, or unnecessary roughness. When I mentioned the barbarism of scrubbing mornings, I did not, of course, mean to imply that a ward should not be kept scrupulously clean, but that all ward-floors should by rights be polished. Polished floors can be cleaned far more easily and efficiently than plain boards, and with far less bustle and confusion. They look much better also, and have only fallen into discredit in those hospitals where they have been laid down without a proper arrangement being made for having them regularly and properly polished."

(*To be continued.*)

